# FET PET Radiomics for Differentiating Pseudoprogression from Early Tumor Progression in Glioma Patients Post-Chemoradiation

**DOI:** 10.3390/cancers12123835

**Published:** 2020-12-18

**Authors:** Philipp Lohmann, Mai A. Elahmadawy, Robin Gutsche, Jan-Michael Werner, Elena K. Bauer, Garry Ceccon, Martin Kocher, Christoph W. Lerche, Marion Rapp, Gereon R. Fink, Nadim J. Shah, Karl-Josef Langen, Norbert Galldiks

**Affiliations:** 1 Institute of Neuroscience and Medicine (INM-3, -4, -11), Research Center Juelich, 52425 Juelich, Germany; mai.elahmadawy@nci.cu.edu.eg (M.A.E.); r.gutsche@fz-juelich.de (R.G.); m.kocher@fz-juelich.de (M.K.); c.lerche@fz-juelich.de (C.W.L.); g.r.fink@fz-juelich.de (G.R.F.); n.j.shah@fz-juelich.de (N.J.S.); k.j.langen@fz-juelich.de (K.-J.L.); n.galldiks@fz-juelich.de (N.G.); 2 Department of Stereotaxy and Functional Neurosurgery, Faculty of Medicine and University Hospital Cologne, University of Cologne, 50937 Cologne, Germany; 3 Department of Nuclear Medicine, National Cancer Institute (NCI), Cairo University, 11796 Cairo, Egypt; 4 RWTH Aachen University, 52062 Aachen, Germany; 5 Department of Neurology, Faculty of Medicine and University Hospital Cologne, University of Cologne, 50937 Cologne, Germany; jan-michael.werner@uk-koeln.de (J.-M.W.); elena.bauer@uk-koeln.de (E.K.B.); garry.ceccon@uk-koeln.de (G.C.); 6 Center for Integrated Oncology (CIO), Universities Aachen, Bonn, Duesseldorf and Cologne, 50937 Cologne, Germany; 7 Department of Neurosurgery, University of Duesseldorf, 40255 Duesseldorf, Germany; marion.rapp@med.uni-duesseldorf.de; 8 Department of Neurology, University Hospital RWTH Aachen, 52074 Aachen, Germany; 9 JARA-BRAIN-Translational Medicine, 52074 Aachen, Germany; 10 Department of Nuclear Medicine, University Hospital RWTH Aachen, 52074 Aachen, Germany; 11 Center for Integrated Oncology (CIO), Universities Aachen, Bonn, Duesseldorf and Cologne, 52074 Aachen, Germany

**Keywords:** machine learning, artificial intelligence, textural features, amino acid PET

## Abstract

**Simple Summary:**

Following chemoradiation with alkylating agents in glioma patients, structural magnetic resonance imaging (MRI) may suggest tumor progression which subsequently improves during the course of the disease without any treatment change. This phenomenon has been termed pseudoprogression. Despite advances in medical imaging, a reliable diagnosis of pseudoprogression remains a challenging task. Radiomics is a subdiscipline of artificial intelligence and allows the identification and extraction of imaging features from various routine imaging modalities. These features can be used for the generation of mathematical models to improve diagnostics in patients with brain tumors. The present study highlights the potential of radiomics obtained from amino acid positron emission tomography (PET) for the diagnosis of pseudoprogression. In 34 patients with suspicious MRI early after chemoradiation completion, our radiomics model correctly identified all patients with pseudoprogression.

**Abstract:**

Currently, a reliable diagnostic test for differentiating pseudoprogression from early tumor progression is lacking. We explored the potential of O-(2-[^18^F]fluoroethyl)-L-tyrosine (FET) positron emission tomography (PET) radiomics for this clinically important task. Thirty-four patients (isocitrate dehydrogenase (IDH)-wildtype glioblastoma, 94%) with progressive magnetic resonance imaging (MRI) changes according to the Response Assessment in Neuro-Oncology (RANO) criteria within the first 12 weeks after completing temozolomide chemoradiation underwent a dynamic FET PET scan. Static and dynamic FET PET parameters were calculated. For radiomics analysis, the number of datasets was increased to 102 using data augmentation. After randomly assigning patients to a training and test dataset, 944 features were calculated on unfiltered and filtered images. The number of features for model generation was limited to four to avoid data overfitting. Eighteen patients were diagnosed with early tumor progression, and 16 patients had pseudoprogression. The FET PET radiomics model correctly diagnosed pseudoprogression in all test cohort patients (sensitivity, 100%; negative predictive value, 100%). In contrast, the diagnostic performance of the best FET PET parameter (TBR_max_) was lower (sensitivity, 81%; negative predictive value, 80%). The results suggest that FET PET radiomics helps diagnose patients with pseudoprogression with a high diagnostic performance. Given the clinical significance, further studies are warranted.

## 1. Introduction

Pseudoprogression is a phenomenon characterized by progressive enhancing lesions on structural magnetic resonance imaging (MRI) unrelated to tumor progression. These findings either remain stable or ultimately regress on follow-up MRI without any change of treatment [1]. Pseudoprogression typically occurs within the first 12 weeks after completing radiotherapy in patients with glioblastoma [2,3,4]. This time-dependent definition has also been incorporated into the criteria defined by the Response Assessment in Neuro-Oncology (RANO) Working Group [2]. Importantly, overlooking pseudoprogression bears the risk of a premature termination of an effective treatment, potentially negatively impacting survival.

Pseudoprogression is caused by local tissue reactions following chemoradiation, resulting in a partial breakdown of the blood-brain barrier, thereby imitating tumor progression [4,5]. A reliable diagnosis of pseudoprogression based on contrast-enhanced MRI alone remains challenging [6,7]. To overcome this problem, several advanced imaging modalities, such as perfusion- and diffusion-weighted MR imaging (PWI, DWI), MR spectroscopy, and static and dynamic parameters derived from amino acid positron emission tomography (PET), were evaluated [6,8,9,10]. However, due to the long acquisition time, dynamic amino acid PET cannot be easily implemented in clinical routine [11,12,13]. Furthermore, the comparability of PWI, DWI, and MR spectroscopy results is hampered by the variable levels of standardization across many centers in terms of data acquisition and post-processing [8]. Therefore, a diagnostic test for reliably differentiating pseudoprogression from early tumor progression is lacking.

More recently, radiomics has gained increasing attention in medical imaging and also in the field of neurooncology [14]. As a subdiscipline of artificial intelligence and machine learning, radiomics aims at the computation, identification, and extraction of quantitative imaging features from routinely acquired imaging data and the generation of predictive or prognostic models. Predominantly, MR studies have already demonstrated the potential of radiomics for the prediction of molecular markers, such as the isocitrate dehydrogenase (IDH) genotype [15,16,17] or O^6^-methylguanine-DNA methyltransferase (MGMT) promoter methylation [18,19,20], the assessment of prognosis in patients with newly diagnosed glioblastoma [21], and for the differentiation of radiation-induced changes predominantly after radiosurgery from local tumor relapse in patients with brain metastases [22,23,24]. 

Concerning the differentiation of pseudoprogression from tumor progression, initial studies suggested the potential of MRI radiomics for this challenging task [25,26]. Although its spatial resolution is inferior, and its availability is lower, the acceptance and the application of PET in patients with brain tumors have steadily increased over the past years [27,28]. In contrast to the widely used PET tracer 2-deoxy-2-[^18^F]fluoro-D-glucose, the uptake of radiolabeled amino acids in normal brain tissue is low. The high uptake of amino acid PET tracers in both gliomas and brain metastases is predominantly caused by an increased transport of large neutral amino acids using the L-type amino acid transporter system (LAT1 and 2) [29,30,31,32,33]. Hence, brain tumors can be depicted with a high tumor-to-background contrast [6]. Moreover, the uptake of amino acid tracers is independent of the blood-brain barrier [34]. In neurooncology, amino acid PET has demonstrated its additional diagnostic value in patients with glioblastoma for various indications, such as biopsy guidance and treatment planning [35,36,37,38], response assessment [39,40], and prognostication in patients with newly diagnosed glioma [41,42].

The RANO Working Group and the European Association of Neuro-Oncology (EANO) advocate the use of PET imaging in patients with brain tumors in addition to MRI at all stages of the disease [43,44]. Initial studies demonstrated that amino acid PET radiomics using the tracer O-(2-[^18^F]fluoroethyl)-L-tyrosine (FET), either as a single modality or in combination with anatomical MRI, encodes valuable diagnostic information in patients with glioma for the prediction of the IDH genotype, the differentiation between radiation injury and recurrent brain metastases, and the assessment of prognosis in newly diagnosed glioma patients [17,24,45]. Thus, the goal of the present study was to evaluate the potential of FET PET radiomics for the diagnosis of pseudoprogression.

## 2. Patients and Methods

### 2.1. Patients

Thirty-four patients (mean age, 57 ± 12 years; age range, 24–79 years; *n* = 13 females) with newly diagnosed, histomolecularly characterized glioma according to the current WHO classification of Tumors of the Central Nervous System [46] (*n* = 32 WHO grade IV glioblastoma, IDH wildtype; *n* = 1 WHO grade IV glioblastoma, IDH mutant; *n* = 1 WHO grade III anaplastic astrocytoma, IDH wildtype) were retrospectively included in the study. Table 1 presents details of the patient cohort.

Macroscopic resections were performed in 26 patients, and eight patients received biopsies. Based on the early postoperative MRI performed within the first 48 h, 13 resections were rated as complete, and 13 were incomplete. According to the RANO criteria [2], in all patients, the first MRI within 12 weeks after completion of chemoradiation with temozolomide (according to European Organization for Research and Treatment of Cancer (EORTC) trial 22981/26981 [47]) was suspicious for tumor progression, i.e., enlargement of contrast-enhancing lesions of more than 25% or new contrast-enhancing lesions inside the radiation field. Subsequently, all patients were additionally investigated using dynamic FET PET within the following 7–10 days. Progression-free survival (PFS) was defined as the time interval from initial histomolecular diagnosis until diagnosis of the first tumor progression or recurrence. Overall survival time (OS) was defined as the time interval from initial histomolecular diagnosis to the date of death. 

### 2.2. Ethics Statement

The study adheres to the standards established in the declaration of Helsinki. The local ethics committees approved the retrospective analysis of the data. All patients had provided written informed consent before each FET PET investigation.

### 2.3. Determination of the IDH Genotype and MGMT Promoter Methylation Status

The IDH mutation status was assessed by the IDH1R132H protein expression level evaluated by immunohistochemistry [48,49]. If immunostaining was negative, IDH was directly sequenced. The 1p/19q co-deletion status was analyzed by fluorescence in situ hybridization [50]. For evaluating the MGMT promoter methylation status using a methylation-specific polymerase chain reaction (PCR) [51], DNA extraction was performed from formalin-fixed and paraffin-embedded tissue samples containing tumor tissue with a histologically estimated tumor cell content of more than 80%. 

### 2.4. Diagnosis of Pseudoprogression and Early Tumor Progression

The criteria described by Young and colleagues [52] were used for the diagnosis of pseudoprogression and early tumor progression. Compared to the initial MRI, progressive enhancing or newly appearing lesions within 12 weeks after completion of chemoradiation with temozolomide were classified as either pseudoprogression or early tumor progression based on pathology after repeated tumor resection or biopsy, or, by clinicoradiological follow-up assessed every 8–12 weeks. In the cases without neuropathological confirmation, pseudoprogression was diagnosed if (i) the imaging findings regressed or remained stable during follow-up MRI, (ii) no change in treatment was required for at least six months after completion of chemoradiation with concurrent temozolomide, and (iii) the patient was clinically stable. Early tumor progression was diagnosed if MRI changes were associated with clinical deterioration and prompted a change in treatment.

### 2.5. FET PET Imaging

The amino acid FET was produced and applied as described previously [53,54]. All patients underwent a dynamic FET PET scan from 0 to 40 min post-injection of 3 MBq of FET per kg body weight. All patients were measured on a stand-alone PET scanner (ECAT EXACT HR+, Siemens Medical Systems, Inc., Erlangen, Germany) in 3D mode (32 rings, axial field of view, 15.5 cm; spatial resolution, 5 mm full-width at half maximum) [55]. The reconstructed dynamic dataset consisted of 14 time frames (5 × 1 min; 5 × 3 min; 4 × 5 min) with a reconstructed voxel size of 2.0 × 2.0 × 2.4 mm^3^. A transmission scan (duration, 10 min) using three rotating line sources (^68^Ge/^68^Ga) was used for attenuation correction. Before iterative ordered subset expectation maximization (OSEM) image reconstruction (16 subsets, 6 iterations), data were corrected for dead time, random and scattered coincidences. 

### 2.6. Evaluation of Static and Dynamic FET PET Parameters

The standardized uptake value (SUV) was used to normalize the FET uptake by dividing the radioactivity in the tissue by the radioactivity injected per gram of body weight. A spherical volume-of-interest (VOI) of constant size (diameter, 30 mm; volume, 14 mL) was positioned in normal-appearing brain tissue, including grey and white matter, in the contralesional hemisphere. A three-dimensional auto-contouring process using a tumor-to-brain ratio (TBR) of 1.6 or more was used for segmenting the tumor volume in the summed PET images from 20–40 min post-injection. This threshold is based on a biopsy-controlled study in which this value separated best between vital tumor and healthy brain parenchyma in FET PET [56]. The mean TBR (TBR_mean_) was calculated by dividing the mean SUV of the tumor VOI by the mean SUV of the background VOI. The maximum TBR (TBR_max_) was calculated by dividing the mean SUV of a spherical VOI (diameter, 16 mm; volume, 2 mL) centered on the voxel with the maximum tumor uptake by the mean SUV of the background VOI [57]. 

The dynamic FET parameters TTP (time in minutes from the beginning of the dynamic acquisition up to the maximum SUV of the tumor) and slope (slope of the linear regression from 20–40 min post-injection expressed in change of SUV per hour) were extracted by the application of the 2 mL spherical VOI centered on the voxel with the maximum uptake to the entire dynamic FET PET dataset. In cases with steadily increasing FET uptake without identifiable maximum uptake, the end of the dynamic PET acquisition was defined as TTP. The background VOI described above was used to generate the time-activity curve (TAC) of the unaffected brain tissue as reference. All processing steps were performed using the software PMOD (version 4.1, PMOD Technologies Ltd., Zurich, Switzerland).

### 2.7. Image Pre-Processing and Radiomics Feature Extraction

Before further processing, the number of available datasets for the radiomics analysis was increased using data augmentation, i.e., three different types of tumor segmentations were used for feature extraction [58]. Besides the conventional FET PET tumor VOIs based on a TBR of 1.6 or more described above, two other sets of tumor VOIs were created using a 10% higher TBR of 1.8 and a 10% lower TBR of 1.4, respectively. Thereby, three different segmentations for each patient were created, and the number of available datasets for feature extraction and model generation was increased from 34 to 102. Before feature extraction, patients were randomly assigned to a training dataset for model training and validation and a test dataset for final model evaluation in a ratio of 70/30, with a balanced distribution of pseudoprogression and early tumor progression diagnoses.

Feature extraction was performed with the open-source Python package pyradiomics (version, 3.0) [59]. No spatial resampling of the PET images was performed. Absolute intensity resampling was performed using a bin width of 0.15, i.e., 64 bins between 0 and 10 SUV. Absolute resampling is recommended for PET studies as it mostly removes high correlations between texture features and metabolic volume that usually occur after relative resampling [60,61]. On the original image, 107 features were calculated for each VOI, including 18 first-order statistics, 14 shape features, 24 features from the grey level co-occurrence matrix (GLCM), 16 features from the grey level run length matrix (GLRLM), 16 features from the grey level size-zone matrix (GLSZM), 5 features from the neighborhood grey level different matrix (NGLDM), and 14 from the grey level dependence matrix (GLDM). A detailed mathematical description of each feature is available in the pyradiomics documentation. Furthermore, high-pass filters using the Laplacian-of-Gaussian image filter (LoG; sigma, 0.5), as well as the discrete 3-dimensional wavelet transformation with the ‘coifl’ wavelet and reconstruction of the higher spatial frequency content in all directions resulting in 8 different wavelet decompositions (images) were applied, and all features (except the shape features) were also calculated on the filtered images. The LoG filter and the wavelet transformation enhance the edges of images and make the feature extraction process more sensitive to small-scale changes of tissue properties [21,62,63]. 

In total, 944 features were calculated for each VOI (107 features on the original image, 93 features on the LoG-filtered image, and 744 features on the wavelet-transformed images (93 features on each of the 8 different wavelet decompositions).

### 2.8. Feature Selection

The calculation of large numbers of features on a limited number of patients poses a specific risk of overfitting and misclassifying the data. Consequently, to reduce the number of features and identify a useful and restricted subset of features for differentiating early tumor progression and pseudoprogression, recursive feature elimination using random forest classifiers was performed. To further reduce the risk of overfitting, 5-fold cross-validation was performed during model generation, and the maximum number of features for a model generation was restricted to 4, according to published recommendations [64,65].

### 2.9. Model Generation and Validation

The best performing machine learning model using the selected features was identified by the Python automated machine learning Tree-based Pipeline Optimization Tool (TPOT) that uses genetic programming to optimize machine learning pipelines [66]. Several different machine learning models with different hyperparameters were applied to the training dataset, and the model with the highest accuracy for differentiating between pseudoprogression and early tumor progression after 5-fold cross-validation was considered best.

### 2.10. Model Testing

**Figure 1:**
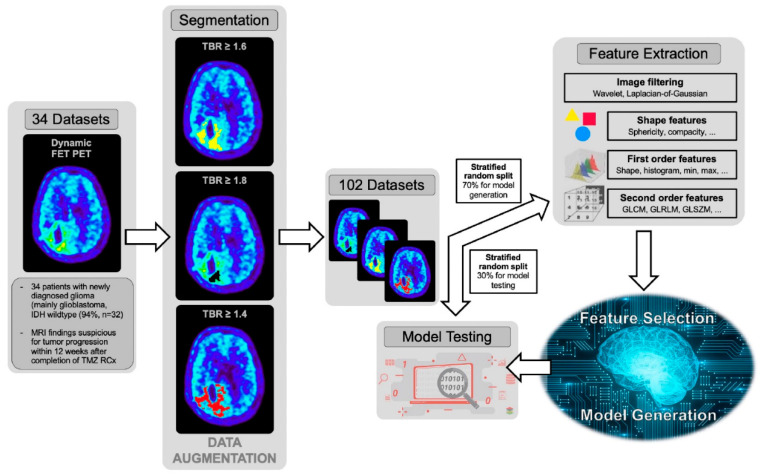
Radiomics workflow—Dynamic O-(2-[^18^F]fluoroethyl)-L-tyrosine (FET) positron emission tomography (PET) measurements of 34 patients with newly diagnosed glioma (mainly glioblastoma, isocitrate dehydrogenase (IDH) wildtype (94%, *n* = 32) and magnetic resonance imaging (MRI) findings suspicious for tumor progression within 12 weeks after completion of chemoradiation with temozolomide were included in the study. Three different tumor segmentations were used to increase the number of datasets for model generation and evaluation from 34 to 102. Before feature extraction, a stratified random split of data in a ratio of 70/30 was performed. On the training datasets (*n* = 72), 107 features were calculated. Furthermore, images were filtered using the Laplacian-of-Gaussian (LoG) and the discrete 3-dimensional wavelet transformation to enhance edges in the images. In total, 944 features were calculated for each patient. Feature selection was performed by recursive feature elimination using the random forest classifiers to avoid overfitting. Furthermore, the number of features for the final model was limited to four. The best performing model on the training data was finally applied to the independent test dataset (*n* = 30).

The best performing model in the training data was applied to the test dataset, which was not involved in model training or validation and, therefore, represents an independent dataset for the evaluation of the robustness and generalizability of the model. The radiomics workflow is presented in Figure 1.

### 2.11. Statistical Evaluation

Descriptive statistics are provided as mean and standard deviation or as median and range. The Mann–Whitney-U test was used for intergroup comparison. Survival analysis was performed using the log-rank test. The static and dynamic PET parameters’ diagnostic performance and combinations were assessed using receiver operating characteristic (ROC) analysis. The cut-off was considered optimal when the product of specificity and sensitivity reached its maximum. The average of the hold-out predictions from the 5-fold cross-validation in each of the resampling iterations was used to evaluate the machine learning models used on the training data. The model performances in the training and the test dataset were evaluated by ROC analysis. Fisher’s exact test for 2 × 2 contingency tables was applied for statistical evaluation of the parameters. *p*-values of 0.05 or less were considered statistically significant. Statistical analyses were performed using SPSS (SPSS Statistics 24, IBM, New York, USA), Microsoft Excel (Excel:Mac 2020, Version 16.35, Microsoft, Redmond, WA, USA), and SciPy [67] (version 1.4.1) for Python (version 3.7.6).

## 3. Results

### 3.1. Pseudoprogression and Early Tumor Progression 

The mean time interval between completion of radiotherapy with concomitant temozolomide and the first MRI was 7 ± 3 weeks (median time, 8 weeks; range, 1–12 weeks). In all 34 patients, imaging findings on the initial MRI suggested tumor progression. Eighteen patients were diagnosed with early tumor progression, and 16 patients (47%) had pseudoprogression (Table 1). Diagnoses were based on histomolecular confirmation in 9 patients (26%) or clinicoradiological follow-up in the remaining 25 patients (74%). Patients with pseudoprogression demonstrated a significantly longer median PFS than patients with early tumor progression (11 vs. 5 months; *p* < 0.001). Additionally, pseudoprogression was associated with a significantly longer median OS (21 vs. 10 months; *p* = 0.010). 

The MGMT promoter methylation status was available for 32 patients (94%). Twelve patients (35%) had tumors with a methylated MGMT promoter, and 20 patients (59%) had tumors with unmethylated MGMT promoter. There was no statistical difference between the presence of the MGMT promoter methylation in the group of patients with pseudoprogression and early tumor progression (*p* = 0.411). 

### 3.2. Group Comparison of Static and Dynamic FET PET Parameters

The static FET PET parameters TBR_mean_ and TBR_max_ were significantly higher in the group of patients with early tumor progression compared to pseudoprogression (TBR_mean_, 2.1 ± 0.2 vs. 1.9 ± 0.1; *p* = 0.025; TBR_max_, 2.6 ± 0.6 vs. 2.0 ± 0.3; *p* = 0.003). The dynamic parameters TTP and slope were not significantly different between the groups. 

### 3.3. ROC Analysis of Static and Dynamic FET PET Parameters

The static FET PET parameters TBR_mean_ and TBR_max_ yielded diagnostic accuracies of 68% (AUC, 0.73; sensitivity, 75%; specificity, 61%; cut-off, 1.95; *p* = 0.045) and 74% (AUC, 0.79; sensitivity, 81%; specificity, 67%; cut-off, 2.25; *p* = 0.007), respectively. The results from the dynamic FET PET parameters TTP and slope were not statistically significant. The highest diagnostic accuracy was achieved by combining TBR_mean_, TBR_max_, and the dynamic FET PET parameter TTP (accuracy, 79%; sensitivity, 69%; specificity, 89%; *p* < 0.001). Other combinations of two or more static and dynamic PET parameters did not further increase the diagnostic performance. Further details are provided in Table 2 and Table 3.

### 3.4. Performance of Machine Learning Models in the Training Dataset

According to the recursive feature elimination, the four most important parameters for differentiating early tumor progression from pseudoprogression were the shape feature *MajorAxisLength*, the two first-order features *Energy* and *Maximum*, and the second-order feature *Size-zone non-uniformity* calculated from the GLSZM. All features used in the final model were extracted from the unfiltered FET PET images. 

The final model used a random forest classifier and achieved an accuracy in the training data of 86% (AUC, 0.74; sensitivity, 82%; specificity, 90%; *p* < 0.001). Further details on the performance of the machine learning model are provided in Table 4. 

### 3.5. Performance of the Machine Learning Model in the Test Dataset

The final machine learning model showed 70% accuracy in the test dataset and correctly identified all patients with pseudoprogression (AUC, 0.74; sensitivity, 100%; specificity, 40%; *p* = 0.017). Further details on the performance of the machine learning model are provided in Table 4. 

## 4. Discussion

The main finding of the present pilot study is that our radiomics model based on routinely acquired static FET PET scans diagnosed pseudoprogression in all patients of the test cohort correctly. Thus, the fully automated application of the proposed radiomics model based on amino acid PET has the potential to serve as a diagnostic tool for pseudoprogression in patients with equivocal MRI findings after completion of temozolomide chemoradiation. Although our model showed a lower overall diagnostic performance in the test dataset with an AUC of 0.74, the sensitivity and negative predictive value were 100%. 

Several studies in glioma patients have evaluated the value of amino acid PET for differentiating early tumor progression from pseudoprogression and reported high diagnostic accuracies in the range of 79–94% [11,12,13,68]. Importantly, these studies combined static and dynamic FET PET parameters, which requires a costly and time-consuming dynamic PET acquisition of at least 40 min, hampering clinical implementation. In contrast, the radiomics analysis in our study uses only imaging features extracted from a 20 min static PET acquisition, which is less laborious, more economical, and already part of clinical routine in many neurooncological centers in Europe. Furthermore, the application of the developed radiomics model does not require specialized hardware and can be performed entirely automatically on a conventional computer in a few seconds. Of note, amino acid PET radiomics should not be considered a stand-alone method, but rather as an additional diagnostic information source based on routinely acquired imaging data.

Up to now, studies evaluating the potential of radiomics for differentiating early tumor progression and pseudoprogression were predominantly based on advanced MRI. For example, Kim and colleagues [25] combined structural MRI with DWI and PWI and generated a radiomics model using 12 features that could diagnose pseudoprogression in a test cohort with an AUC of 0.85. Elshafeey and co-workers [26] built a classifier using 60 radiomic features from multicentric PWI data that could diagnose pseudoprogression in a test dataset with a high diagnostic accuracy (AUC, 0.89). However, due to the large number of parameters used in these models, the interpretation is challenging, limiting its clinical acceptance. 

In contrast, our radiomics model based on FET PET utilizes only four parameters, which may ameliorate its interpretation and clinical acceptance. The clinical acceptance of radiomics and its subsequent clinical translation depends mainly on the diagnostic benefit. In our opinion, easy implementation into clinical routine and improved model interpretability, which often appears complex at first glance, may contribute significantly to the clinical translation. Therefore, we concentrated on models with a small number of parameters, which makes an interpretation more straightforward and lowers the risk of overfitting considerably. 

**Figure 2:**
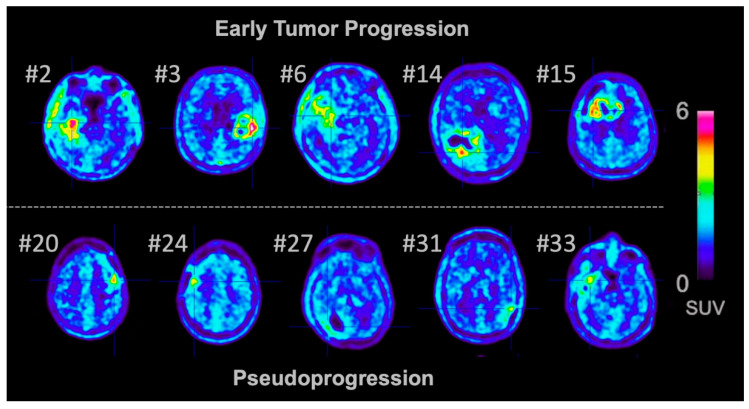
Representative O-(2-[^18^F]fluoroethyl)-L-tyrosine (FET) positron emission tomography (PET) images of glioblastoma patients with early tumor progression (top row) and pseudoprogression (bottom row). Patients with pseudoprogression showed a slightly lower and more homogenous FET uptake, whereas patients with early tumor progression showed a more heterogenous FET uptake. This visual impression was also reflected by the identified radiomics parameters.

The features used in the final model of our study were (i) the shape feature *MajorAxisLength*, which yields the most extensive axis length of the VOI-enclosing ellipsoid; (ii) the histogram-derived feature *Energy,* which is a measure of the magnitude of voxel values; (iii) the histogram-derived feature *Maximum*, which represents the maximum value in the VOI; (iv) and the second-order feature *Size-zone non-uniformity* from the GLSZM, which measures the variability of size-zone volumes in the image with a lower value indicating more homogeneity. All features in the final model were extracted from the unfiltered images, and, interestingly, patients with early tumor progression showed a more heterogeneous FET uptake than patients with pseudoprogression (Figure 2). Similarly, a previous study reported that patients with recurrent brain metastases following stereotactic radiosurgery also exhibit a more heterogenous FET uptake compared to patients with radiation-induced changes [24]. To further elucidate this unclear observation, a direct comparison of radiomics features with histomolecular parameters is warranted. 

Several limitations of the study need to be discussed. The presented cohort is small, but, as we strictly adhere to the time-dependent definition of pseudoprogression, the risk of misclassification was considerably reduced and contributed to a reasonably homogenous patient collective. Additionally, using slightly different tumor segmentations, the number of datasets for the radiomics analysis, model generation, and evaluation could be increased and allowed a meaningful machine learning workflow. Furthermore, multiple segmentations limit the extent of bias introduced by the segmentation variability, thus enabling robust features to be identified [69,70,71]. 

Model robustness and generalizability were demonstrated in an independent test dataset, and the risk of overfitting was minimized by reducing the number of features to four and performing cross-validation during model training. Nevertheless, a multicenter dataset is desirable for model evaluation. 

## 5. Conclusions

In summary, the presented FET PET radiomics model correctly diagnosed all patients with pseudoprogression in an independent test dataset without the need for costly and time-consuming dynamic FET PET scans. Thereby, this approach justifies routine clinical application. Despite the promising performance of the developed radiomics model in the test dataset, further validation of the developed model in a large multicentric dataset is necessary. Since some studies have shown a synergistic effect by combining PET and MRI radiomics [24,72], the combination of FET PET radiomics with structural, as well as advanced, MRI radiomics should also be further investigated, especially in the light of the growing number of hybrid PET/MR scanners. This pilot study results are promising and suggest an important role for FET PET radiomics in neurooncology.

## Figures and Tables

**Table 1:** Overview of the patient cohort.

#	Gender	Age	Initial Diagnosis	IDH Genotype	MGMT Promoter Methylation	EoR	Weeks Since Last Radiation	FU Diagnosis	Confirmation of FU Diagnosis	PFS [Months]	OS [Months]	TBR_mean_	TBR_max_	TTP [min]	Slope [SUV/h]
**1**	m	58	GBM	wt	unmeth	PR	6	EP	Histomolecular	4	9	1.8	1.9	12.5	−0.4
**2**	m	61	GBM	wt	unmeth	CR	4	EP	Histomolecular	5	5 *	2.1	2.4	37.5	0.4
**3**	m	52	GBM	wt	unmeth	PR	4	EP	Histomolecular	5	15	2.2	2.8	37.5	1.1
**4**	m	69	GBM	wt	unmeth	PR	8	EP	Histomolecular	6	12	1.9	1.9	22.5	0.3
**5**	f	43	GBM	wt	unmeth	CR	8	EP	Follow-up	5	24 *	2.0	2.5	32.5	0.3
**6**	m	52	GBM	wt	unmeth	CR	7	EP	Follow-up	5	16	1.9	2.3	32.5	0.6
**7**	m	72	GBM	wt	unmeth	B	5	EP	Follow-up	4	7	2.2	3.7	18.5	−0.8
**8**	m	51	GBM	wt	meth	PR	6	EP	Histomolecular	5	22 *	2.5	3.9	12.5	−1.9
**9**	m	44	GBM	wt	unmeth	CR	3	EP	Follow-up	3	9	1.9	2.6	37.5	1.3
**10**	m	61	GBM	wt	unmeth	B	8	EP	Follow-up	5	6 *	2.2	2.8	27.5	−0.2
**11**	m	57	GBM	wt	unmeth	B	5	EP	Follow-up	2	2 *	2.1	2.8	18.5	−1.5
**12**	m	42	GBM	wt	meth	CR	12	EP	Follow-up	5	34	2.1	2.5	15.5	0.1
**13**	m	79	GBM	wt	n.a.	CR	12	EP	Histomolecular	n.a.	6 *	1.9	1.9	22.5	0.1
**14**	m	76	GBM	wt	meth	B	4	EP	Follow-up	4	6	2.0	2.6	18.5	−0.2
**15**	f	52	GBM	wt	meth	PR	8	EP	Follow-up	5	15 *	2.0	2.0	27.5	0.2
**16**	m	54	GBM	wt	unmeth	PR	8	EP	Follow-up	8	10	1.8	1.8	32.5	0.9
**17**	m	69	GBM	wt	meth	B	1	EP	Follow-up	4	4	1.9	2.1	27.5	0.9
**18**	f	52	GBM	wt	unmeth	PR	4	EP	Follow-up	8	11 *	2.6	3.5	37.5	1.0
**19**	f	71	GBM	wt	meth	PR	3	PSP	Histomolecular	12	21	1.8	1.8	37.5	0.4
**20**	f	76	GBM	wt	meth	CR	8	PSP	Histomolecular	20	20 *	1.9	2.2	22.5	−0.3
**21**	f	58	GBM	wt	unmeth	PR	8	PSP	Follow-up	24	38	2.0	2.1	22.5	−0.1
**22**	m	50	GBM	wt	unmeth	CR	4	PSP	Follow-up	16	23	1.9	2.0	37.5	1.0
**23**	m	34	GBM	wt	meth	PR	8	PSP	Follow-up	60	65 *	1.8	1.8	37.5	0.4
**24**	f	48	GBM	wt	n.a.	CR	8	PSP	Histomolecular	n.a.	14 *	1.9	1.9	27.5	−0.5
**25**	f	64	GBM	wt	meth	CR	4	PSP	Follow-up	n.a.	50	1.7	1.7	37.5	1.2
**26**	f	66	GBM	wt	unmeth	B	12	PSP	Follow-up	10	12	2.3	3.0	22.5	−0.7
**27**	m	66	GBM	wt	meth	B	12	PSP	Follow-up	16	23 *	2.2	2.5	27.5	0.4
**28**	f	49	GBM	wt	unmeth	PR	6	PSP	Follow-up	10	11 *	1.9	2.2	27.5	0.0
**29**	f	24	AA	wt	unmeth	B	11	PSP	Follow-up	8	12 *	2.1	2.4	18.5	−0.5
**30**	f	51	GBM	wt	unmeth	CR	8	PSP	Follow-up	8	18	1.8	1.8	27.5	0.6
**31**	m	44	GBM	wt	unmeth	PR	10	PSP	Follow-up	6	12	1.9	1.9	32.5	0.2
**32**	m	65	GBM	wt	meth	CR	6	PSP	Follow-up	11	13 *	1.8	1.8	37.5	0.9
**33**	f	68	GBM	wt	unmeth	PR	9	PSP	Follow-up	10	10 *	1.8	1.8	37.5	0.9
**34**	m	48	GBM	mut	meth	CR	6	PSP	Follow-up	24 *	24 *	1.8	1.8	37.5	0.5

AA = anaplastic astrocytoma; B = biopsy; CR = complete resection; EOR = extent of resection; EP = early tumor progression; f = female; FU = follow-up; GBM = glioblastoma; IDH = isocitrate dehydrogenase; m = male; meth = MGMT promoter methylated; MGMT = O^6^-methylguanine-DNA methyltransferase; mut = mutant; n.a. = not available; OS = overall survival; PFS = progression-free survival; PR = partial resection; PSP = pseudoprogression; TBR_max_, TBR_mean_ = maximum and mean tumor-to-brain ratio; TTP = time-to-peak; wt = wildtype; * = censored.

**Table 2:** Classification results of conventional FET PET parameters for differentiating pseudoprogression from early tumor progression.

Parameter	TBR_mean_	TBR_max_	TTP	Slope
Sensitivity	75%	81%	75%	56%
Specificity	61%	67%	44%	61%
PPV	63%	68%	55%	56%
NPV	73%	80%	67%	61%
FNR	25%	19%	25%	44%
FPR	39%	33%	56%	39%
Accuracy	68%	74%	59%	59%
F1 Score	0.69	0.74	0.63	0.56
MCC	0.36	0.48	0.20	0.17
AUC	0.73	0.79	0.61	0.55
Cut-off	≤ 1.95	≤ 2.25	≥ 25 min	≥ 0.3 SUV/h
*p*-value ^#^	0.045	0.007	0.297	0.492

AUC = area under the receiver operating characteristic curve; FNR = false negative rate; FPR = false positive rate; MCC = Matthews correlation coefficient; NPV = negative predictive value; PPV = positive predictive value; TBR_max_, TBR_mean_ = maximum and mean tumor-to-brain ratio; TTP = time-to-peak; # = Fisher’s exact test.

**Table 3:** Classification results of conventional FET PET parameter combinations for differentiating pseudoprogression from early tumor progression.

Parameter Combinations ^§^	TBR_mean_ + TBR_max_	TBR_mean_ + TTP	TBR_mean_ + Slope	TBR_max_ + TTP	TBR_max_ + Slope	TTP + Slope	TBR_mean_ + TBR_max_ + TTP
Sensitivity	75%	69%	50%	69%	50%	56%	69%
Specificity	72%	78%	78%	83%	89%	61%	89%
PPV	71%	73%	67%	79%	80%	56%	85%
NPV	76%	74%	64%	75%	67%	61%	76%
FNR	25%	31%	50%	31%	50%	44%	31%
FPR	28%	22%	22%	17%	11%	39%	11%
Accuracy	74%	74%	65%	76%	71%	59%	79%
F1 Score	0.73	0.71	0.57	0.73	0.62	0.56	0.76
MCC	0.47	0.47	0.29	0.53	0.43	0.17	0.59
*p*-value ^#^	0.015	0.014	0.151	0.005	0.023	0.492	0.001

FNR = false negative rate; FPR = false positive rate; MCC = Matthews correlation coefficient; NPV = negative predictive value; PPV = positive predictive value; TBR_max_, TBR_mean_ = maximum and mean tumor-to-brain ratio; TTP = time-to-peak; § = other combinations did not further increase the model performance; # = Fisher’s exact test.

**Table 4:** Classification results of the machine learning model for differentiating pseudoprogression from early tumor progression.

	Training Data	Test Data
Number of datasets	72	30
Feature selection method	RFE (random forest)	
Number of features	4	
Classifier	Random forest	
Sensitivity	82%	100%
Specificity	90%	40%
PPV	87%	63%
NPV	85%	100%
FNR	18%	0%
FPR	10%	60%
Accuracy	86%	70%
F1 Score	0.84	0.77
MCC	0.72	0.50
AUC	0.74	0.74
*p*-value ^#^	< 0.001	0.017

AUC = area under the receiver operating characteristic curve; FNR = false negative rate; FPR = false positive rate; MCC = Matthews correlation coefficient; NPV = negative predictive value; PPV = positive predictive value; RFE = recursive feature elimination; **#** = Fisher’s exact test.
